# Modeling the Potential Impact of Seasonal and Inactive Multi-Aquifer Wells on Contaminant Movement to Public Water-Supply Wells^1^

**DOI:** 10.1111/j.1752-1688.2011.00526.x

**Published:** 2011-06

**Authors:** RL Johnson, BR Clark, MK Landon, LJ Kauffman, SM Eberts

**Keywords:** groundwater hydrology, simulation, drinking water, nonpoint source pollution, point source pollution, source water protection, water supply

## Abstract

Wells screened across multiple aquifers can provide pathways for the movement of surprisingly large volumes of groundwater to confined aquifers used for public water supply (PWS). Using a simple numerical model, we examine the impact of several pumping scenarios on leakage from an unconfined aquifer to a confined aquifer and conclude that a single inactive multi-aquifer well can contribute nearly 10% of total PWS well flow over a wide range of pumping rates. This leakage can occur even when the multi-aquifer well is more than a kilometer from the PWS well. The contribution from multi-aquifer wells may be greater under conditions where seasonal pumping (e.g., irrigation) creates large, widespread downward hydraulic gradients between aquifers. Under those conditions, water can continue to leak down a multi-aquifer well from an unconfined aquifer to a confined aquifer even when those multi-aquifer wells are actively pumped. An important implication is that, if an unconfined aquifer is contaminated, multi-aquifer wells can increase the vulnerability of a confined-aquifer PWS well.

## Introduction

Public water-supply (PWS) wells are often sited in confined aquifers because confined systems generally offer more protection from near-surface sources of groundwater contamination than do unconfined aquifers. However, even aquifers beneath laterally extensive confining units can be vulnerable to contamination if natural or man-made preferential flow pathways through the confining unit exist (Santi *et al.*, 2006). This is particularly the case for multi-aquifer wells; that is, wells screened across confining units. Seasonal irrigation wells are important in this context because, in many areas, these wells have been and continue to be installed with long screened intervals that connect multiple aquifers. As a result, they may provide a direct connection between unconfined aquifers and deeper confined aquifers.

At the same time, high-volume pumping (e.g., irrigation) in confined aquifers frequently creates significant downward hydraulic gradients between the confined and overlying unconfined aquifers (Chen *et al.*, 2005). These downward hydraulic gradients can encompass multiple square-kilometer areas. During periods of heavy pumping, it is not uncommon for vertical hydraulic head differences between aquifers to be several meters of water or more (e.g., Chen *et al.*, 2005; Clark *et al.*, 2008). Thus, if wells are screened in both confined and unconfined aquifers, substantial downward flow to the confined aquifer can occur. If shallow groundwater in the vicinity of these wells is contaminated with natural or anthropogenic contaminants, including pathogens, then relatively young contaminated water can quickly reach the confined aquifer without the benefit of attenuation processes otherwise provided by the overlying confining unit.

The goal of the work presented here is to show that a simple numerical model can provide site-specific insight into the potential impact of multi-aquifer wells that are seasonally pumped (e.g., irrigation) and/or inactive (e.g., unused, abandoned and improperly decommissioned wells, test holes) on a confined-aquifer PWS well. As seasonally pumped wells frequently play a controlling role in overall aquifer stress (e.g., Clark *et al.*, 2008), understanding their effect on the movement of potentially contaminated water to PWS wells in confined aquifers also is an important objective of the work presented here.

### Previous Studies

The impact of inactive multi-aquifer wells on confined aquifers has been examined previously using both mathematical modeling and field studies. Silliman and Higgins (1990) developed analytical solutions for steady-state flow between aquifers through an open well. More recently, Zinn and Konikow (2007) conducted a numerical modeling study on the effects of wellbores with long screened or open intervals on the transport of water and solutes. Their results demonstrate that inactive wells completed in multiple aquifer layers can transport water and solutes rapidly over large vertical distances. Results were highly dependent on assumed flow conditions, but in all simulations the inactive wells substantially altered the groundwater age distribution in the aquifer. When pumping in proximity to an inactive well was simulated, the inactive well served as a conduit for downward flow of recently recharged (i.e., “young”) water to deeper parts of the aquifer, even where the inactive well had served as a conduit for upward flow of older water prior to pumping. Likewise, an earlier modeling study by Lacombe *et al.* (1995) indicated that contaminants from an upper aquifer can rapidly migrate downward along an open or sediment-filled borehole and result in an extensive contaminant plume in a lower aquifer. At the regional scale, wells screened across multiple aquifers have been shown to influence the overall water balance of confined aquifers (Williamson *et al.*, 1989; Hanson *et al.*, 2004; Hart *et al.*, 2006). For example, Hanson *et al.* (2004) used simulations to estimate that wellbore flow represented 19% of total groundwater flow between layered aquifers in the Santa Clara Valley, California.

### Delineation of Wellhead Protection Areas in Relation to Multi-aquifer Wells

Since passage of the Amendments to the Safe Drinking Water Act (SDWA) in 1986, an extensive literature on delineation of wellhead protection areas (WHPAs) has developed, including “Guidelines for delineation of wellhead protection areas” (USEPA, 1987) and “Well-head protection strategies for confined-aquifer settings” (USEPA, 1991). These and other publications use a range of terminologies to describe wellhead protection areas, including one that will be used here. USEPA (1987) defined the “zone of transport” (ZOT) as the 2D projection to land surface defined by “an isochrone indicating the time necessary for water or a conservative contaminant to reach the well.”

In the context of contaminant transport to PWS wells, a 40-year time of travel is recommended for differentiating semiconfined and highly confined conditions (USEPA, 1991; Savoca *et al.*, 2002). As an approximation of the area over which multi-aquifer wells would be anticipated to impact a confined-aquifer PWS well, an estimate of the 40-year ZOT can be calculated from the volume of water pumped over 40 years divided by the thickness of the transmissive zone and divided by the porosity of the aquifer [i.e., the so called “cylinder” or “volumetric flow” calculation (USEPA, 1987)].

Numerical models can simulate more realistic conceptual models of groundwater flow than what is simulated with the cylinder model. However, standard approaches for using numerical models to estimate ZOTs (e.g., USEPA, 1987) do not take multi-aquifer wells into account. This is partly because commonly used numerical modeling programs (e.g., MODFLOW, Harbaugh *et al.*, 2000) have not had the capability of simulating wells with screens extending across multiple layers while allowing water to simultaneously flow into and out of different portions of the well screen. This changed for MODFLOW with development of the Multi-Node Well (MNW) package (Halford and Hanson, 2002).

**FIGURE 1:**
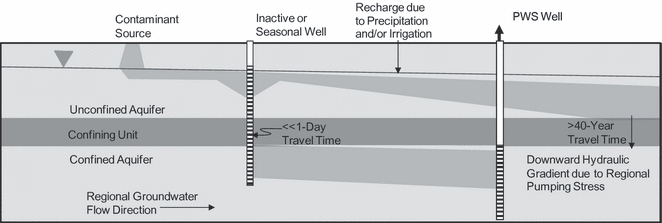
Conceptual Drawing Showing the Impact of an Inactive Multi-aquifer Well on Contaminant Movement to a Public Water-Supply Well.

In cases where anthropogenic contaminants have impacted a PWS well, it is typically difficult to document whether or not multi-aquifer wells within the ZOT for the well have played a substantial role. At the same time, it is difficult to determine *a priori* the likelihood that such contamination will occur, even if multi-aquifer wells are known to be present in a given study area. Johnson *et al.* (2000) used a probabilistic approach to estimate the potential impact of releases to shallow groundwater on PWS wells. The focus of that work was on releases from underground storage tanks near the water table. Their numerical modeling analysis indicated that, for most scenarios, there was a pumping rate threshold below which contamination would not be drawn down to the PWS well screen. Not surprisingly, that analysis showed that a confining unit between the water table and the PWS well screen substantially increased that threshold. In the work discussed here, it is shown that the threshold may be reduced or eliminated by rapid transport (leaking) from an overlying unconfined aquifer into a confined aquifer because of the presence of one or more multi-aquifer wells (Figure 1) and downward hydraulic gradients across the confining unit due to regional pumping (e.g., for irrigation and or PWS).

To demonstrate that simple models can be used to assess the potential impact of multi-aquifer wells on confined-aquifer PWS wells, we use a well-studied example from York, Nebraska (Clark *et al.*, 2008; Landon *et al.*, 2008). At that location, U.S. Geological Survey researchers identified 62 multi-aquifer production wells (primarily irrigation wells) and test holes in an ≈62-km^2^ area – a density of approximately 1 multi-aquifer well per km^2^. Interspersed among the multi-aquifer wells were 58 confined-aquifer wells. Heavy pumping from the confined aquifer put a significant hydraulic stress on the aquifer and resulted in downward hydraulic gradients of more than 10 m throughout the area during irrigation seasons (Landon *et al.*, 2008).

Using aquifer and pumping conditions similar to those in York, a volumetric calculation of the 40-year ZOT for a confined-aquifer PWS well gives an area of ≈12 km^2^ and suggests that ≈12 multi-aquifer wells could lie within the ZOT (Table 1, top). Assuming an annual average regional downward hydraulic gradient of 3 m, the volume of water that would be estimated to leak down an inactive multi-aquifer well for the York example is ≈260 m^3^/day (Table 1, bottom). Taken together, the total volume leaked down the 12 wells potentially within the 40-year ZOT (≈3,120 m^3^/day) is significantly greater than the typical pumping rates for PWS wells near York (400-2,000 m^3^/day). Thus, if one or more inactive, multi-aquifer wells were contaminated, there could be a potentially significant impact on a PWS well. Of course, both the presence of multi-aquifer wells and seasonal pumping would affect the ZOT for a PWS well, but their combined effect cannot be estimated using the simple calculations in Table 1. However, as shown below, a simple numerical model can be used to take these interactions into account.

**TABLE 1:** Calculation of a 40-Year Zone of Transport, Number of Multi-aquifer Wells Potentially Impacting a Public Water-Supply Well, and Volumetric Flow Down an Inactive Multi-aquifer Well: Example for Confined-Aquifer Setting Similar to York, Nebraska

Daily pumping rate	1,200 m^3^/day
Total volume pumped over 40 years	17,520,000 m^3^
Aquifer thickness	10 m
Effective aquifer porosity	0.15
40-year zone of transport	17,520,000 m^3^/10 m/0.15
	≈12 km^2^
Estimated density of multi-aquifer wells	1.0 well/km^2^
Number of multi-aquifer wells potentially impacting the PWS well	12 km^2^ × 1.0 well/km^2^
	12 wells
*Q* = 2π*K*_C_*b*_C_(*H*_U_ − *H*_C_)/ln(*r*_C_/*r*_W_) where, *K*_C_ is the hydraulic conductivity of the confined aquifer (≈9 m/day), *b*_C_ is the thickness of the confined aquifer (≈10.5 m), (*H*_U_ − *H*_C_) is the hydraulic head difference across the confining unit (3 m), *r*_C_ is the radius at which the hydraulic head is measured in the confined aquifer (assumed here to be 100 m, at which point the confined head will likely not be significantly influenced by flow down the leaking well), and *r*_W_ is the radius of the well (0.1 m, assumed) (Silliman and Higgins, 1990)^1^	
Volumetric flow down inactive multi-aquifer well	
≈2π × 9 × 10.5 × 3/ln(100/0.1)	
≈260 m^3^/day	
≈0.06 million gallons per day	

Note: PWS, public water supply.

1 Assumptions inherent in this calculation include that the hydraulic head in the well equals that in the unconfined aquifer and the well is fully screened across the confined aquifer. The flow calculated by this equation may be significantly reduced if there is resistance to flow through the well (see, for example, the discussion in Halford and Hanson, 2002).

## Methods

**FIGURE 2:**
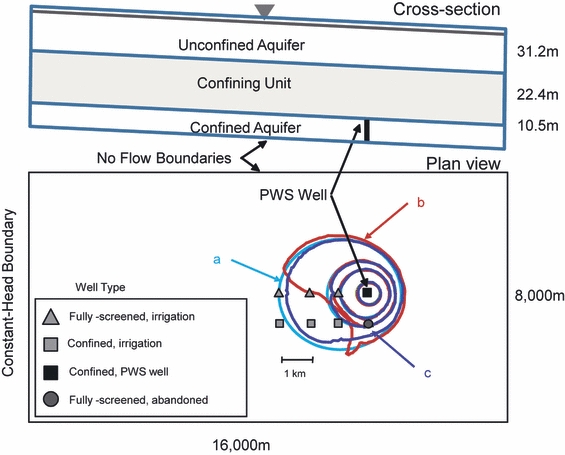
Map Showing Simplified Numerical Model Domain and Simulated Effects of Multi-aquifer Wells on Flow to a Confined-Aquifer Public Water-Supply (PWS) Well. The extent of the simulated 1-, 5-, 10-, and 40-year zones of transport (ZOT) in the confined aquifer for the PWS well are shown for the (a) PWS-well-only scenario, (b) seasonal scenario, and (c) inactive scenario. The pumping rate at the PWS well for these model results was 1,200 m^3^/day.

Schematic plan and cross-section views for a simple numerical model of a confined-aquifer setting similar to York, Nebraska are shown in Figure 2. The model domain is 16 km long by 8 km wide by ≈63 m thick and is composed of 11 layers with grid blocks that are 40 m on a side. To simulate the regional groundwater flow, “constant-head” boundaries were used on the “short” sides – 74.9 and 59.4 m for the left and right sides respectively. “No-flow” boundaries were used on the “long” sides of the model domain because (1) the effects of pumping the PWS well extended only ≈2 km beyond the well, (2) these boundaries are approximately parallel to the regional groundwater flow direction, and (3) it is reasonable to assume that regionally extensive irrigation pumping is similar inside and outside the model domain and that the model boundaries are, therefore, lines of symmetry. Water-table conditions were assumed in the unconfined aquifer, and no-flow conditions were assumed along the bottom boundary. Recharge was applied at 0.0001 m/day, except in the vicinity of irrigation wells for the four months each year when water was applied. For recharge during irrigation, the application rate was 0.00025 m/day to a 1-km^2^ rectangle centered on each irrigation well.

In the vicinity of the PWS well, the confined aquifer is represented by three layers at the bottom of the model with an overall thickness of 10.5 m and with horizontal hydraulic conductivity values of 6.1-15 m/day (Table 2, adapted from Clark *et al.*, 2008). The confined aquifer is overlain by a 22.4-m-thick confining unit with a horizontal hydraulic conductivity of 0.03 m/day. A 31.2-m-thick unconfined aquifer with horizontal hydraulic conductivity values from 12 to 55 m/day overlies the confining unit. A vertical anisotropy value of 30 was used for all layers in these simulations.

**TABLE 2:** Model Parameters Used in the Simplified Numerical Model (adapted from Clark *et al.*, 2008)

Layer	Thickness (m)	Horizontal *K* (m/day)	Vertical *K* (m/day)	Porosity	Specific Storage	Specific Yield	Representative Aquifer or Unit
1	4.2	55	1.5	0.15	0.00001	0.15	Unconfined aquifer
2	18.6	55	1.5	0.15	0.00001	0.15	Unconfined aquifer
3	4.2	34	1.1	0.15	0.00001	0.15	Unconfined aquifer
4	4.2	12	0.4	0.15	0.00001	0.15	Unconfined aquifer
5	5.6	0.03	0.001	0.35	0.00001		Confining unit
6	5.6	0.03	0.001	0.35	0.00001		Confining unit
7	5.6	0.03	0.001	0.35	0.00001		Confining unit
8	5.6	0.03	0.001	0.35	0.00001		Confining unit
9	3.5	6.1	0.2	0.15	0.00001		Confined aquifer
10	3.5	15	0.5	0.15	0.00001		Confined aquifer
11	3.5	6.1	0.2	0.15	0.00001		Confined aquifer

The model domain described above was used to examine three different scenarios. First, a single PWS well and steady recharge were simulated. No additional wells were included in this simulation (“PWS-well-only” scenario). Second, the PWS-well-only scenario was simulated with the addition of six seasonal irrigation wells and one abandoned well (“seasonal” scenario) (see Figure 2, plan view, for well configuration). Third, the seasonal scenario was simulated without any pumping for irrigation (“inactive” scenario). To assess sensitivity of the results to PWS well pumping, three different PWS well pumping rates were examined in each scenario (400, 1,200, and 2,000 m^3^/day or approximately 0.1, 0.3, and 0.5 million gallons per day). For the seasonal scenario, the irrigation wells were pumped at a rate of 1,200 m^3^/day per well for a four-month period and then were inactive for the remaining eight months of each year. Three of the six irrigation wells were fully screened across the model domain, and three were screened only in the confined aquifer (Figure 2). All of the wells were simulated using MODFLOW 2000 (Harbaugh *et al.*, 2000) and the MNW package (Halford and Hanson, 2002).

For each model scenario and pumping rate, ZOTs were estimated using backward particle tracking in MODPATH (Pollock, 1994). This was accomplished by starting particles in an array on each face of the cells representing the PWS well. Particles were tracked backwards within the confined aquifer until they reached a source of water (in this case, MNW cells that leaked water) or until the desired travel time (1, 5, 10, or 40 years) was reached. The ZOTs were then calculated using the maximum horizontal extent of the tracked particles within the confined aquifer for each travel time.

To determine the fractions of leaked water reaching the PWS well within 40 years or less travel time, forward particle tracking in MODPATH was used. Particles were started on the faces of model cells where there was a source of flow from an MNW. The particles were released during the first year of the simulation and tracked until they reached a pumping well or the end of the 40-year simulation period. The cells representing the PWS wells were strong sinks, so a volume of water could be assigned to each tracked particle by dividing the flow across the model-cell face where a particle was started by the number of particles started on that face. The percentage of water pumped by the PWS well that originated as leakage down a given up-gradient multi-aquifer well was computed by summing the volumes associated with the particles released in the leaking MNW cells that reached the PWS well, dividing the computed volume by the volume of water pumped at the PWS well, and multiplying by 100.

## Results and Discussion

**FIGURE 3:**
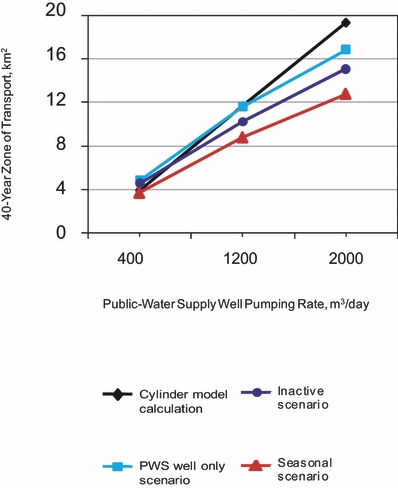
Graph Showing 40-Year Zones of Transport for Various Public Water-Supply (PWS) Well Pumping Rates in the Simplified Numerical Model.

A steady-state simulation for the PWS-well-only scenario at the middle pumping rate (1,200 m^3^/day) produced a 40-year ZOT in the confined aquifer of 11.6 km^2^ (Figures 2 and 3). This value is similar to the simple cylinder model calculation presented in Table 1 (≈12 km^2^). The 40-year ZOT for the seasonal scenario was notably smaller (8.8 km^2^) because much of the water pumped at the PWS well was supplied by leakage down the abandoned well and the nearest multi-aquifer irrigation well (Table 3). The shape of the 40-year ZOT in this scenario was affected by pumping at the confined-aquifer irrigation wells. The size of the 40-year ZOT for the inactive scenario was between those computed for the PWS-well-only and the seasonal scenarios because some of the water pumped at the PWS well was supplied by leakage down multi-aquifer wellbores, but, as discussed below, flow down the multi-aquifer wells for the inactive case was less than for the seasonal case because the downward hydraulic gradients were smaller. The one- and five-year ZOTs for the three different scenarios were essentially the same because the ZOTs did not extend beyond the closest multi-aquifer wells (Figure 2).

**TABLE 3:** Wellbore Leakage Rates and Percents of PWS Well Flow Rates from Multi-aquifer Wells

		PWS Well Pumping Rate (m^3^/day)					
		
		Inactive Scenario (no irrigation pumping)			Seasonal Scenario (seasonal irrigation pumping)		
			
Well	Distance From PWS Well (km)	2,000	1,200	400	2,000	1,200	400
Net wellbore flow rate from unconfined to confined aquifer (m^3^/day)^1^							
Abandoned well	1	153	95	37	195	137	80
Multi-aquifer irrigation well	1	149	92	36	176	120	64
Multi-aquifer irrigation well	2	49	32	16	86	70	54
Multi-aquifer irrigation well	3	23	17	11	52	46	40
All wells (abandoned + three irrigation wells)		374	237	100	510	373	237
Percent of water pumped at the PWS well from multi-aquifer wellbore leakage 40 years after leakage began							
Abandoned well	1	8	8	0	10	11	6
Multi-aquifer irrigation well	1	7	8	9	9	10	13
Multi-aquifer irrigation well	2	2	2	2	2	0	0
Multi-aquifer irrigation well	3	1	0	0	0	0	0
All wells (abandoned + three irrigation wells)		17	17	11	20	21	20

Notes: PWS, public water supply; MNW, Multi-Node Well.

1 Inactive scenario values from the MODFLOW MNW QSUM file. Seasonal scenario values derived from the MODFLOW MNW QSUM file; reported rates are averages from the time steps representing the last year of the simulation weighted by the time-step lengths.

**FIGURE 4:**
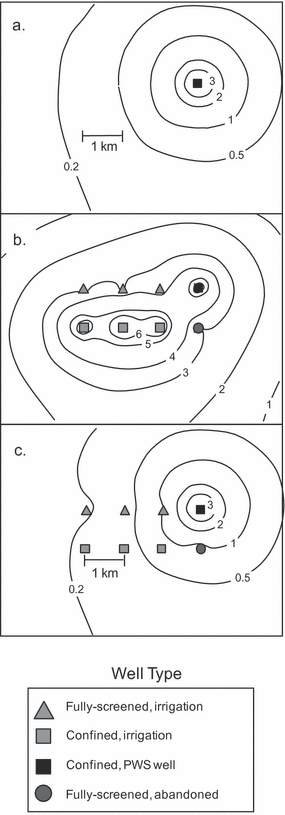
Contours of Hydraulic Head Difference Between the Confined and Unconfined Aquifers (in meters) During Irrigation and Nonirrigation Conditions. Scenarios are (a) PWS-well-only, (b) seasonal, and (c) inactive.

As in many confined aquifer settings developed for water supply, hydraulic head differences were observed across the confining unit in the simulated scenarios. For the PWS-well-only scenario with a pumping rate of 1,200 m^3^/day, differences of a meter or more were observed in the vicinity of the PWS well (Figure 4a). In contrast, when the seasonal wells were active, hydraulic head differences of more than a meter were observed throughout most of the model domain (Figure 4b). When the irrigation wells were present but not pumped (the inactive scenario; Figure 4c), the hydraulic head differences were similar to those in the PWS-well-only scenario.

Differences in the vertical hydraulic gradients among the scenarios resulted in differences in the multi-aquifer wells that contributed flow to the PWS well and the amount of water leaked down the multi-aquifer wells (Table 3). Not all of the leaked water reached the PWS well during the simulations. For the seasonal scenario, however, multi-aquifer wells contributed a total of about 20% of the flow to the PWS well regardless of the pumping rate at the PWS well. This is because the three confined-aquifer irrigation wells collectively had a greater influence on vertical hydraulic gradients – and thus the downward flow of water to the confined aquifer – than did the PWS well. The smaller vertical hydraulic gradients in the inactive scenario resulted in less water leaked down individual multi-aquifer wells. Consequently, the volume of water needed to satisfy the pumping demand at the PWS well had to come from a larger area, and more multi-aquifer wells contributed flow to the PWS well. Unlike in the seasonal scenario, however, the pumping rate at the PWS well in the inactive scenario affected the percentage of leaked water that reached the PWS well. The smallest percentage of leaked water produced by the PWS well (11%) was for the inactive scenario simulation with the lowest pumping rate (400 m^3^/day). Both the inactive and seasonal scenarios demonstrated that nearly 10% of the water produced by a confined-aquifer PWS well can come through a single multi-aquifer well (Table 3), even when the well is a kilometer away from the PWS well.

Interestingly, for the multi-aquifer irrigation wells, water continued to flow from the unconfined aquifer to the confined aquifer even when the wells were actively pumped. For example, the model indicated that if the total flow out of each multi-aquifer irrigation well was 1,200 m^3^/day, then flow from the unconfined aquifer into the irrigation wells ranged from 1,246 to 1,320 m^3^/day (determined near the end of the four-month irrigation period). At the same time, a portion of the water that entered the wells from the unconfined aquifer (46-120 m^3^/day) flowed down the wells and *out* of the lower portions of the same irrigation wells into the confined aquifer. This occurred because the pumping rate at the multi-aquifer irrigation wells was too low to overcome the downward flow of water between aquifers that resulted from the large downward hydraulic gradients established in response to pumping at the confined-aquifer wells.

In the absence of multi-aquifer wells (i.e., the PWS-well-only scenario), travel times associated with water flowing from the unconfined aquifer through the confining unit to the confined aquifer were >40 years. In contrast, the model indicated that if a multi-aquifer well was 1 km from the PWS well and the PWS pumping rate was 1,200 m^3^/day, water moving down the multi-aquifer well could reach the PWS well after ≈5 years of travel. Although the model presented here is conceptually simple and contains far fewer multi-aquifer wells than are reported for York, Nebraska, conclusions drawn from the model results are similar to those reached by Clark *et al.* (2008) based on the more-detailed numerical model constructed for the York area.

In other settings, the impact of multi-aquifer wells could be different. For example, a higher hydraulic conductivity in the confined aquifer for a given hydraulic head difference between aquifers would result in a higher flow rate out of the well and into the confined aquifer. However, given similar boundary conditions, a higher hydraulic conductivity would lead to a smaller hydraulic head difference and therefore a smaller flow rate out of the well. Thus, if the impact of multi-aquifer wells on a PWS well is to be explored using a simple numerical model, the model should be constructed to represent the setting of interest.

## Implications for Protection of Public Water-Supply Wells

Predicting the impact of contaminants on a PWS well due to leaking multi-aquifer wells is, in general, not possible. However, the modeling steps described here can provide site-specific insight into the potential for multi-aquifer wells to affect a confined-aquifer PWS well. First, the ZOT for the PWS well can be estimated to assess whether a multi-aquifer well might present a problem. The ZOT should be defined within the confined aquifer (rather than, for example, only considering water reaching the well from the water table within a specific time period). This is because multi-aquifer wells can reduce or eliminate any protection offered by transport through either the unconfined aquifer or the confining unit. Second, site-specific vertical hydraulic gradient data should be obtained so that the volumetric flow down inactive wells from the unconfined aquifer to the confined aquifer can be estimated using the approach developed by Silliman and Higgins (1990). Third, if the setting of interest has complexities such as numerous inactive or seasonally active multi-aquifer wells, the kind of simple numerical model discussed here can be constructed to better understand the potential contributions of those wells to the PWS well.

Although the focus here has been on volumetric water flow rather than water quality, estimates of volumetric flow through multi-aquifer wells do provide insight into the issue of contamination of PWS wells. The numerical modeling data from this study suggest that, if a leaking multi-aquifer well lies within the ZOT of a PWS well, on the order of 10% of the total PWS flow could come from that well. Stated another way, water produced from the PWS well could be expected to have concentrations that are within a factor of 10 of the concentration leaked into the confined aquifer via the multi-aquifer well. As a result, if a leaky well were contaminated, it is likely that the contaminant concentration would be high enough to impact water quality at the PWS well.

For the simulations presented here, seasonal pumping had a controlling impact on the hydraulic gradient across the confining unit during the irrigation season (i.e., when in operation, irrigation wells – especially those screened in the confined aquifer alone – placed substantial stress on the confined aquifer). An important and unexpected result for the case examined here was that, during irrigation, water levels in the multi-aquifer wells remained above the potentiometric surface for the stressed confined aquifer. As a result, water entering those wells flowed from the unconfined aquifer to the confined aquifer under both inactive and actively pumped conditions. In addition, active pumping of the irrigation wells had the potential to draw contaminants to the irrigation wells through the unconfined aquifer at the same time they continued to leak water from the unconfined aquifer to the confined aquifer.

A national survey of PWS wells concluded that anthropogenic volatile organic contaminants (VOCs) were nearly as likely to be found in water from confined aquifers as from unconfined aquifers (62 *vs.* 67% probability of detection) (Squillace and Moran, 2007). For VOC contamination of a confined aquifer to occur, both downward hydraulic gradients and preferential flow pathways through the confining layer are likely to be necessary. Thus, the Squillace and Moran data suggest that preferential flow pathways may occur frequently. Although multi-aquifer wells represent only one type of preferential flow pathway, the modeling results presented here indicate that, where they exist, multi-aquifer wells could be a major factor contributing contaminants to impacted confined-aquifer PWS wells. As a result, multi-aquifer wells, including those that are seasonally active, can lead to situations that may affect public health.
